# Biosynthesis and Characterization of Cross-Linked Fmoc Peptide-Based Hydrogels for Drug Delivery Applications

**DOI:** 10.3390/gels1020179

**Published:** 2015-10-16

**Authors:** Laura Chronopoulou, Silvia Margheritelli, Yosra Toumia, Gaio Paradossi, Federico Bordi, Simona Sennato, Cleofe Palocci

**Affiliations:** 1 Chemistry Department, University of Rome La Sapienza, Piazzale A. Moro 5, 00185 Rome, Italy; E-Mail: laura.chronopoulou@uniroma1.it; 2 Department of Chemical Science and Technology, University of Rome Tor Vergata, Via della Ricerca Scientifica 1, 00133 Rome, Italy; E-Mails: smargheritelli@yahoo.it (S.M.); toumiayosra@hotmail.com (Y.T.); paradossi@stc.uniroma2.it (G.P.); 3 Department of Physics, University of Rome La Sapienza and Institute for Complex-System (ISC) CNR, UOS Sapienza, P.le Aldo Moro 2, I-00185 Roma, Italy; E-Mails: federico.bordi@roma1.infn.it (F.B.); simona.sennato@roma1.infn.it (S.S.)

**Keywords:** peptide hydrogel, biosynthesis, drug delivery

## Abstract

Recently, scientific and technological interest in the synthesis of novel peptide-based hydrogel materials have grown dramatically. Applications of such materials mostly concern the biomedical field with examples covering sectors such as drug delivery, tissue engineering, and production of scaffolds for cell growth, thanks to their biocompatibility and biodegradability. In this work we synthesized Fmoc-Phe_3_ based hydrogels of different chirality by using a biocatalytic approach. Moreover, we investigated the possibility of employing a crosslinker during the biosynthetic process and we studied and compared some chemico-physical features of both crosslinked and non-crosslinked hydrogels. In particular, we investigated the rheological properties of such materials, as well as their swelling ability, stability in aqueous medium, and their structure by SEM and AFM analysis. Crosslinked and non-crosslinked hydrogels could be formed by this procedure with comparable yields but distinct chemico-physical features. We entrapped dexamethasone within nanopolymeric particles based on PLGA coated or not with chitosan and we embedded these nanoparticles into the hydrogels. Dexamethasone release from such a nanopolymer/hydrogel system was controlled and sustained and dependent on genipin crosslinking degree. The possibility of efficiently coupling a drug delivery system to hydrogel materials seem particularly promising for tissue engineering applications, where the hydrogel could provide cells the necessary support for their growth, while nanoparticles could favor cell growth or differentiation by providing them the necessary bioactive molecules.

## 1. Introduction

Tissue engineering and regenerative medicine are part of an emerging multi- and interdisciplinary field that applies the principles of engineering and life sciences towards the development of biological substitutes [1,2]. Such research fields have the potential to revolutionize the way health and quality of life are improved for millions of people worldwide by restoring, maintaining, or enhancing tissue and organ function. Different elements are believed to be crucial for successful tissue regeneration: stem cells, growth factor, and scaffold. Cells provide the machinery for new tissue growth and differentiation, whereas growth factors and other molecules modulate the cellular activity and provide stimuli for cells to differentiate and support tissue neogenesis.

A three-dimensional template structure for cell growth is provided by scaffolds able to support and facilitate the processes that are critical for tissue regeneration [3]. The nanotechnology approach to scaffold design and synthesis is an emerging area of research and study [4,5], one of the current biggest challenges is to exploit self-assembly processes (the spontaneous organization of matter into specific arrangements) to obtain materials and devices with innovative characteristics and functions, especially for biomedical and biotechnological use [6,7]. The aim is to achieve pre-defined specific, ordered or disordered, structures via the rational design of elementary “building blocks”. In this “bottom-up approach”, the effort is made in the direction of a rational design of the elementary components of the requested structure. Despite the large emphasis on the importance of the bottom-up approach in the production of new materials, up to now research has mostly focused on the synthesis and characterization of novel nanoparticles or of new macromolecules with the potential to self-assemble and, less frequently, on the study of collective structures (micelles, fibers, sheets, or three-dimensional networks, gels) arising from their self-assembly.

Peptide hydrogels are interesting materials that are currently studied for their potential use in biomedical applications [8,9]. Recently, we have reported the lipase-supported synthesis of Fmoc-tripeptides, which occur in an aqueous phase through a reverse hydrolysis reaction [10]. These materials are biocompatible, as well as biodegradable and they possess a very interesting feature, which is their injectability, since the precursors used for their synthesis are liquid at room temperature. The possibility to use such biomaterials as drug delivery vehicles induced many scientists to investigate the possibility of modulating the crosslinking degree of the macromolecular 3D structures by using different crosslinking agents.

Genipin is a natural compound, found in *Gardenia jasminoides* fruit extracts. It has traditionally been used in herbal medicine and as a food dye [11,12]. Genipin is known to be able to act as a crosslinking agent for proteins and aminoacids, affording stable cross-linked products. In particular peptidic hydrogels can exhibit, as a function of their crosslinking degree, different mechanical properties in comparison to the non-crosslinked ones. Moreover, such chemical modification may be able to influence their *in vivo* stability. The mechanism of the genipin crosslinking reaction is still not fully understood, however it involves the formation of genipin dimers that bind amine groups on adjacent proteins [13,14], that give a blue-colored reaction product. Genipin is a particularly interesting cross-linking agent because of its low cytotoxicity, especially if compared with traditional crosslinkers such as glutaraldehyde and epoxy compounds [15]. Moreover, it has been recently reported that the presence of genepin may favor cell adhesion to artificial matrices [16]. For the above reasons, the use of genipin in the preparation of new materials for biomedical applications is highly attractive. So far, it has been used to crosslink polymeric hydrogel-forming materials such as gelatin and fibrin [16,17] or polypeptide hydrogels [18].

In this work, we used genipin for the first time as a crosslinker for Fmoc-tripeptide hydrogels of different chirality, synthesized by a lipase-supported reaction in aqueous phase that we developed in the past [10,19]. We characterized the rheological and chemico-physical properties of the obtained materials and we compared them with those of non-crosslinked ones in order to assess if genipin-mediated crosslinking could provide attractive features to the hydrogels in view of their use in tissue engineering approaches. In fact, such materials may be used as artificial scaffolds for cell growth, an approach that may lead to future applications in tissue engineering. With this objective, we loaded the hydrogels with a model drug, dexamethasone (DXM), and we studied its release kinetics from the different hydrogel materials also by using nanopolymeric vectors based on polylactic-co-glycolic polymers embededded with the Desamethsone (DXM) with the aim to modulate drug release.

## 2. Results and Discussion

### 2.1. Hydrogel Biosynthesis

**Figure 1:**
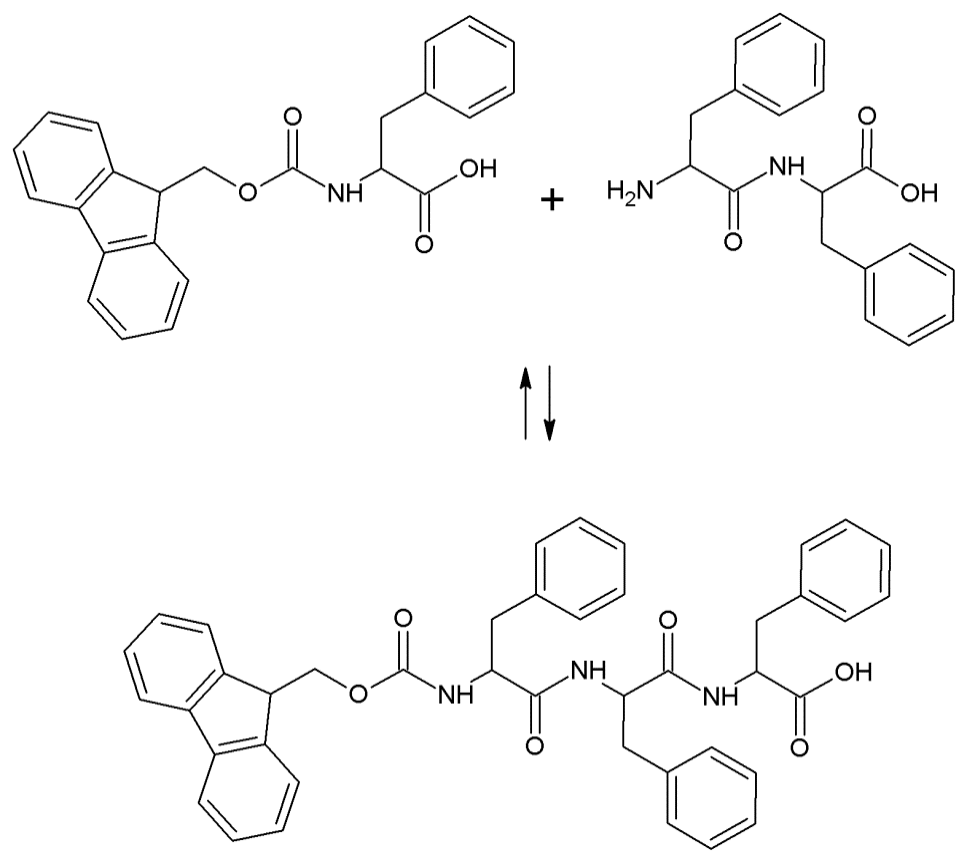
Reaction scheme of FmocFFF or FmocF*F*F* formation.

Both FmocF and FmocF* were used in lipase-catalyzed reversed hydrolysis reactions using respectively FF and F*F* dipeptides, with the formation of a peptide bond between the Fmoc-aminoacid and the dipeptide (Figure 1). The reaction products are FmocFFF and FmocF*F*F* tripeptides. The reaction conditions for such bioconversions have been optimized in previous works [19] and were employed both for non-crosslinked as well as for genipin-crosslinked hydrogels. As far as the crosslinked gels preparation is concerned, different genipin concentrations were used, corresponding to values ranging from 1/2000 to 1/20 with respect to the amount of Fmoc-aminoacid used. All the bioconversions, with and without genipin, afforded self-supporting hydrogels in the employed reaction conditions in about 20 min, as evidenced by the inversion test. Chemically crosslinked gels were more firm to the touch and more easily removable from the glass vials in which they were formed, while gels fabricated without genipin were more easily torn during handling. For genipin-crosslinked hydrogels, a blue color, evidence of the ongoing crosslinking reaction, appeared within a few hours of hydrogel preparation. The reaction yields for all bioconversions were calculated, affording the results shown in Table 1. Such results, obtained for non-crosslinked gels and for gels crosslinked with the highest genipin concentration used, evidence that the presence of genipin did not affect the tripeptide reaction yield.

**Table 1:** Reaction yields of PFL-catalyzed bioconversions. Reaction conditions: 40 μmol of Fmoc-aminoacid and dipeptide, 5 mg of PFL (Pseudomonas fluorescence lipase), 100 μL of 10 mM genipin solution, aqueous phase total volume = 3 mL, pH 7.0, T = 30 °C, reaction time 30 min. Values are the means ± s.d. of three independent experiments.

Material	Reaction Yield (%)
FmocF*F*F*	50 ± 2
Genipin-crosslinked FmocF*F*F*	53 ± 3
FmocFFF	27 ± 2
Genipin-crosslinked FmocFFF	32 ± 2

Preliminary investigations showed that genipin can react both with the dipeptide, that possesses an –NH_2_ group, as well as with the Fmoc-tripeptide, that possesses three –NH groups, but not with the Fmoc-aminoacid. The crosslinking reaction starts while the enzymatic reaction occurs, therefore genipin most probably reacts both with the dipeptide as well as with the Fmoc-tripeptide, whose formation triggers self-assembly and hydrogel formation [10]. Although the presence of genipin in the reaction medium does not influence the reaction yield of tripeptide formation, it could significantly influence the self-assembly and three-dimensional organization of the final product.

### 2.2. Rheological Measurements

**Figure 2:**
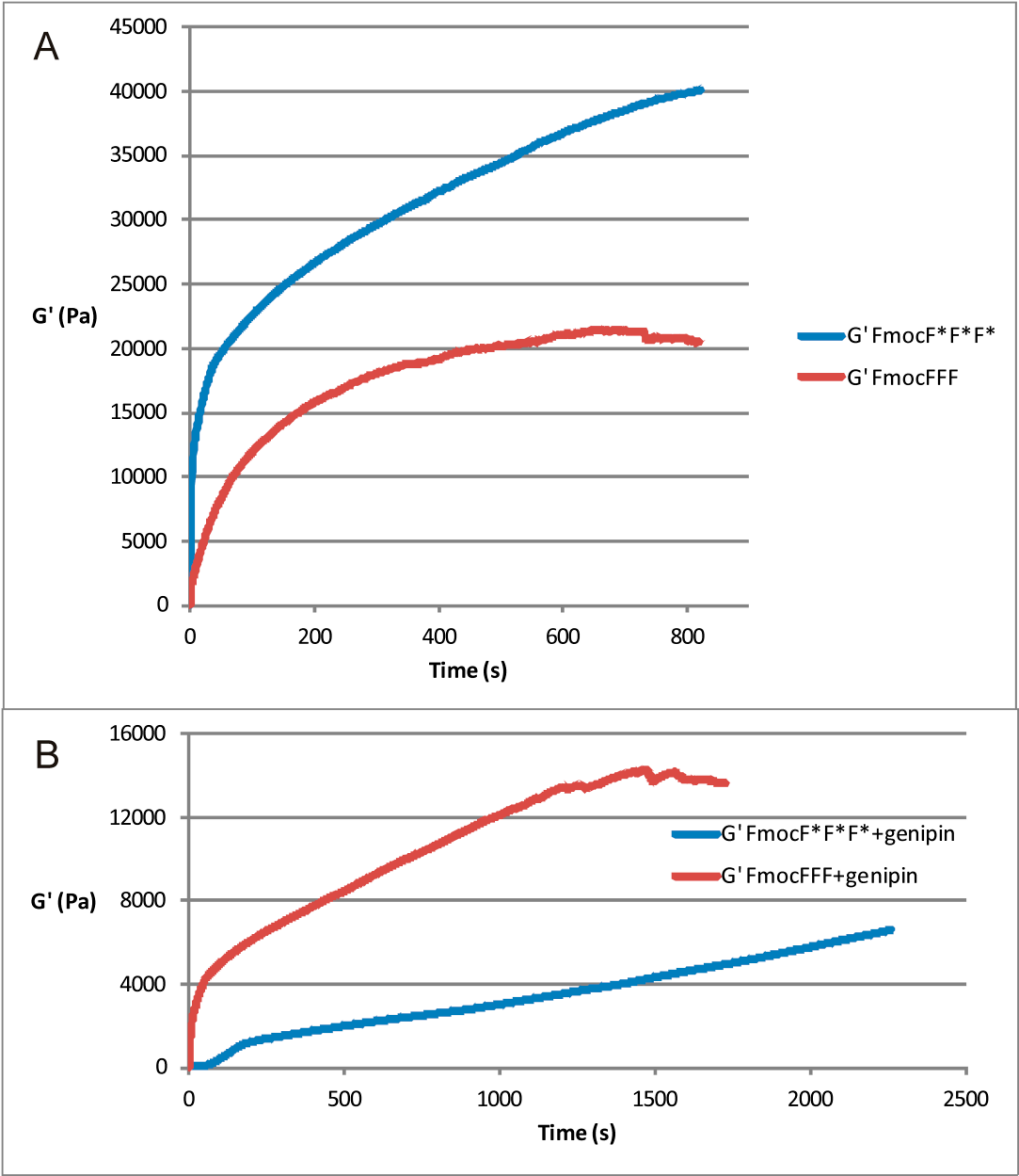
Time evolution of storage modulus (G') of FmocFFF and FmocF*F*F* alone (**A**) and with 10 mM genipin (**B**). T = 30 °C.

As previously reported by the authors [20] the storage modulus, G', is remarkably sensitive to the chirality of the Fmoc-peptide. As shown in Figure 2A, when the polymer network formation involves the D isomer FmocF*F*F*, the value of G' at equilibrium is about twice the value of the storage modulus obtained when the l isomer is used. Such a result can be explained on the basis of an increase in the Fmoc-peptide reaction yield, as well as on the different structure and size of the fibers obtained by using substrates with D-chirality. When genipin is added, the mechanical behavior of the gel is reversed. The mechanical spectra of the genipin-treated hydrogels (Figure 2B) show that the L isomer provides a firmer hydrogel than the D.

**Figure 3:**
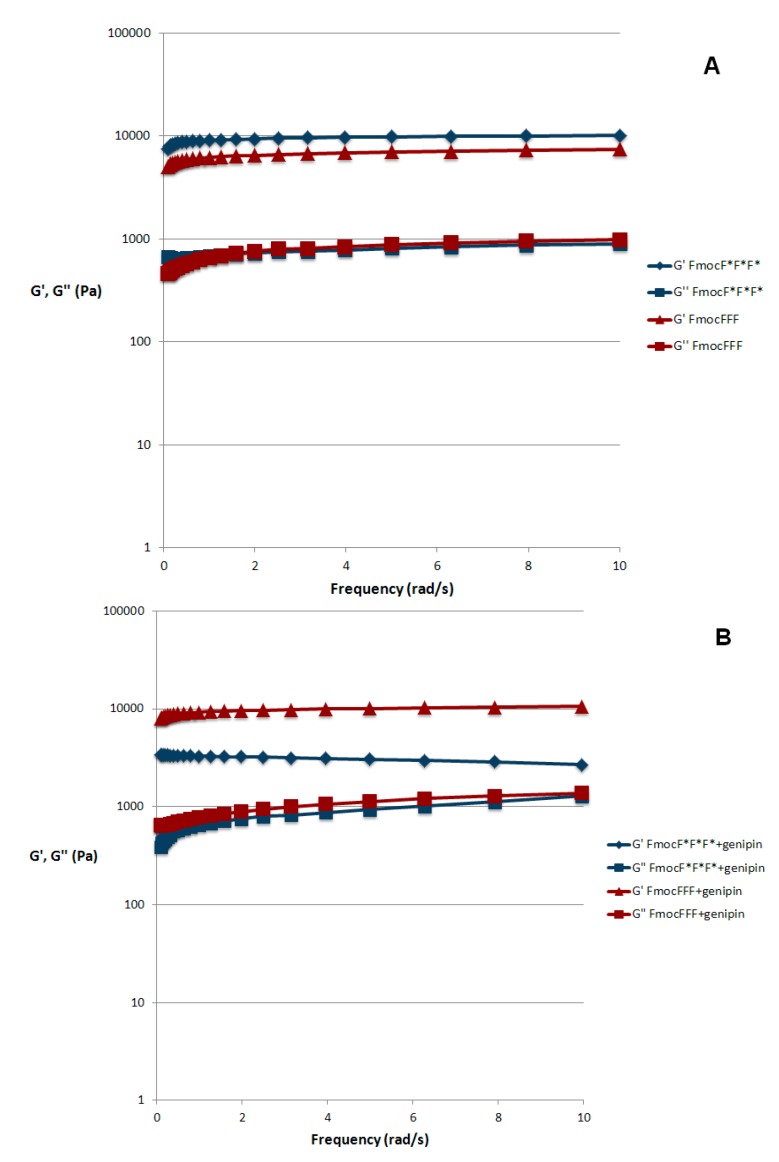
Mechanical spectra of FmocFFF and FmocF*F*F* in the absence of genipin, (**A**), and with 10 mM genipin, (**B**). T = 25 °C. Strain = 1%. Measurements were performed after overnight equilibration.

G' values are known to be directly proportional to the cross-linking density. An evaluation of the initial rate of the cross-linking reaction can provide information on the difference in the formation of the crosslinks in the presence of the two isomers. In the initial part of the kinetics of Fmoc-tripeptides without genipin, where a linear trend is expected, the slope of the curve obtained with d is about the double of the slope registered in the presence of the l isomer. This finding corroborates the results highlighted in the mechanical spectra of Figure 3A.

The presence of genipin lowers the initial growth rate of G' for both isomers. However, consistently with the results of Figure 3B, genipin enhances the G' growth of the l- with respect to the d- isomer. This may reflect a different microscopic organization of the reaction products.

### 2.3. Swelling Ratio and Weight Loss Ratio Measurements

Hydrogel materials designed for biomedical applications will come in contact with biological fluids *in vivo*. Studying *in vitro* the behavior of such materials in the presence of aqueous-based solutions that simulate the *in vivo* environment, such as buffered solutions or Ringer solution, are therefore important and may afford valuable information on the *in vivo* interactions of the hydrogels with the surrounding environment.

**Figure 4:**
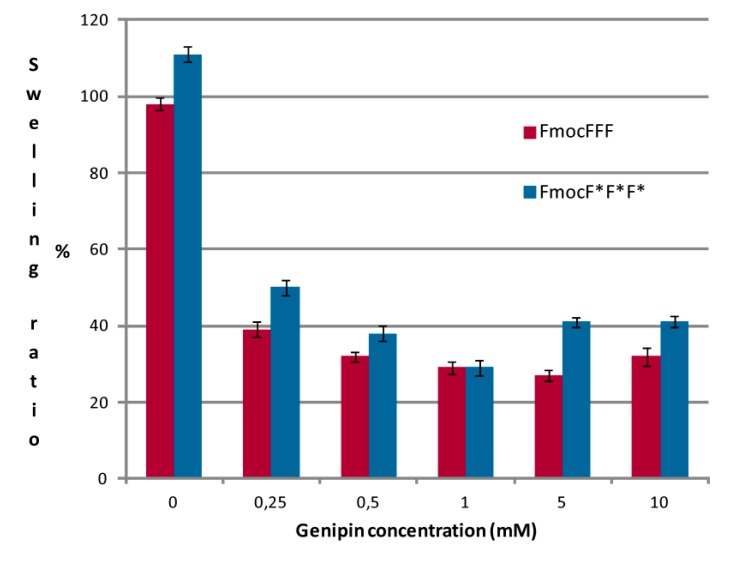
Swelling ratios of peptidic hydrogels with different chirality and genipin concentration in the presence of Ringer solution.

Figure 4 shows the swelling ratios of Fmoc-based hydrogels with different chirality, crosslinked with different genipin concentrations. As reported previously [20], hydrogels with an l chirality show lower swelling abilities than their d counterparts, and this is confirmed also for the genipin-crosslinked materials. Crosslinked hydrogels have lower swelling ratios than non-crosslinked ones (that are around 100%), with no significant differences among the different genipin concentrations used. This behavior may indicate that crosslinked Fmoc-tripeptides preferably interact with the crosslinker or with other tripeptide molecules, rather than with water molecules, therefore their swelling is lower, with values between 27% and 50%.

**Figure 5:**
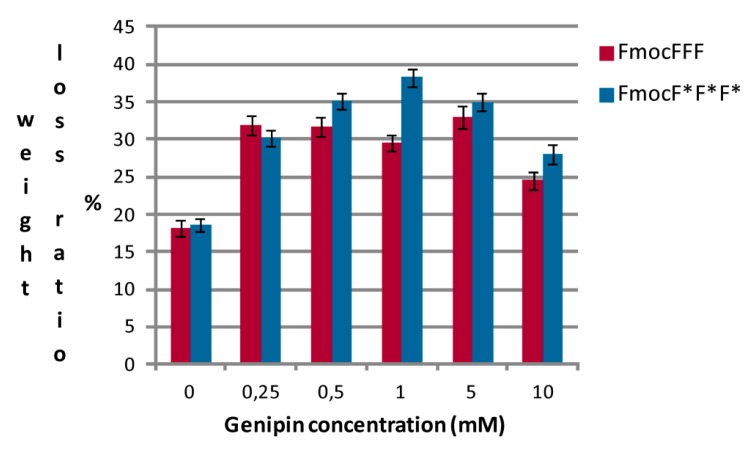
Weight loss ratios in the presence of a Ringer solution of peptidic hydrogels with different chirality and genipin concentration.

The effect of a Ringer solution on the stability of the hydrogels, evaluated through their weight loss ratios, was also studied after an incubation of 30 days, a period of time that can be considered long for such materials. Results are reported in Figure 5. Non-crosslinked hydrogels have a weight loss ratio of about 18%, with no significant differences between the two enantiomers. Cross-linked hydrogels show higher weight loss ratios, between 24% and 38%. There is no clear relation between the amount of crosslinker used and the weight loss ratio of the crosslinked material. Comparing these results with those obtained with different hydrogel materials, such as agar–kappa-carrageenan blend cross-linked with genipin [21], that show weight loss ratios of 15%–40% in approximately two days, we can affirm that all our materials are quite stable.

### 2.4. DXM in Vitro Release Studies

Peptide hydrogels are considered promising materials for biomedical applications, *i.e.*, tissue engineering and tissue regeneration applications. Currently, three elements are considered to be crucial for successful tissue regeneration: stem cells, scaffold and growth factors or other chemicals used for *in vitro* cell differentiation, such as DXM. Therefore, the possibility of employing a hydrogel scaffold as a drug delivery system is a key to the application of such materials in regenerative medicine. With the aim to evaluate the potential of Fmoc-tripeptide hydrogels in this sense, we studied the release kinetics of a model drug into a buffered medium.

**Figure 6:**
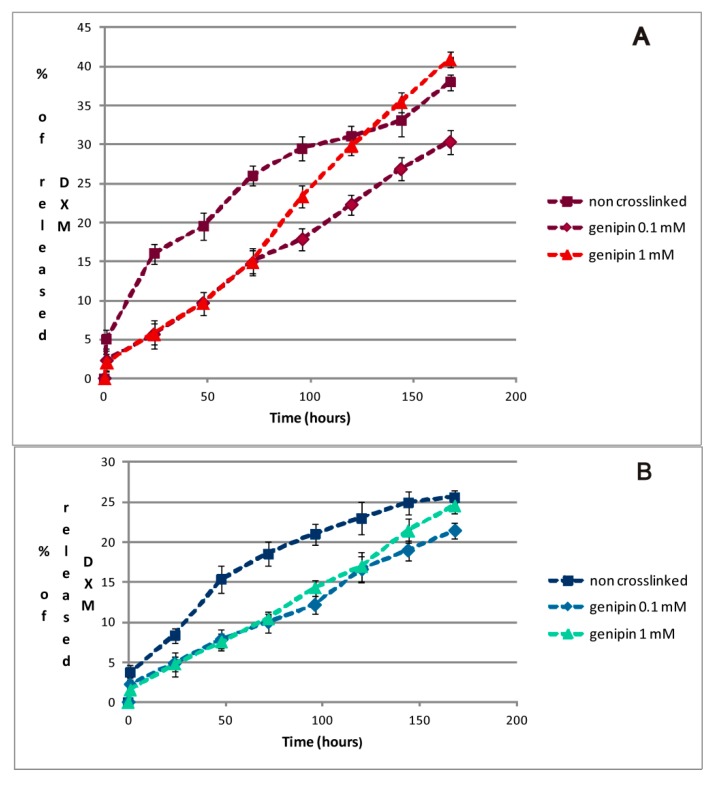
DXM release kinetics from hydrogel matrices with different chirality: FmocFFF (**A**) and FmocF*F*F* (**B**).

Figure 6 shows DXM release kinetics from the peptidic hydrogel matrices of different chirality, both crosslinked with genipin at two different concentrations and non-crosslinked.

DXM is released at a higher rate from hydrogels with l chirality, both crosslinked or non-crosslinked, reaching values between 30% and 40% of the total DXM amount in approximately one week, providing a slow and sustained drug release over time. On the other hand, hydrogels with D chirality release 20%–25% of DXM in the same time frame. This may be due to the different microscopic structure of hydrogels, which is closely linked to their chirality [20]. Overall, in accordance with the respective yields of tripeptide formation, d-amino acid based hydrogels are more “dense” materials, that detain entrapped drugs more than their l counterparts. The presence of genipin-based crosslinking in the hydrogels did not affect significantly the total amount of released DXM, but had an effect on the release kinetics. All the tested materials were able to release the drug in a sustained manner and showed no burst effects, but rather an almost constant release over time.

**Figure 7:**
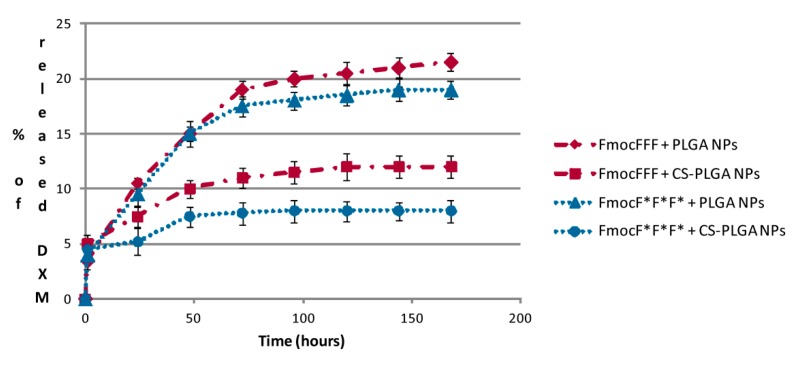
DXM release kinetics from PLGA and CS-PLGA NPs entrapped within FmocFFF and FmocF*F*F* hydrogel matrices.

Moreover, we evaluated DXM release kinetics from polymeric NPs entrapped within FmocFFF and FmocF*F*F* hydrogels (Figure 7). Such hydrogels were chosen because their mechanical properties were the most promising among the materials studied in this work. Also in this case chirality had an influence on drug release, which, as observed for the release of free DXM from Fmoc-based hydrogels discussed above, reached higher values for hydrogels with L chirality. Moreover, for both gels a higher release rate was evidenced for uncoated PLGA NPs in comparison with (CS)-coated NPs. Overall, both formulations afforded a significantly slow and sustained DXM release over time, reaching values between 8% and 20% of released DXM in approximately seven days. Previous studies on NPs alone have already evidenced that DXM is entirely released from such formulations within a couple of hours [22], therefore the presence of the hydrogel matrix is responsible for obtaining a controlled drug release system. Both the gel and PLGA-based NPs seem to be able to interact with DXM and the synergy between such interactions affords its release in a sustained way. In conclusion, the direct encapsulation of DXM into the hydrogel seems to provide a more efficient and sustained release over time, making the system appealing for drug delivery approaches. On the other hand, the slower DXM release provided by the use of NPs, ensuring higher DXM concentrations within the hydrogel matrix, could be interesting in tissue engineering applications, *i.e.*, cells grown within the hydrogel.

### 2.5. SEM and AFM Measurements

**Figure 8:**
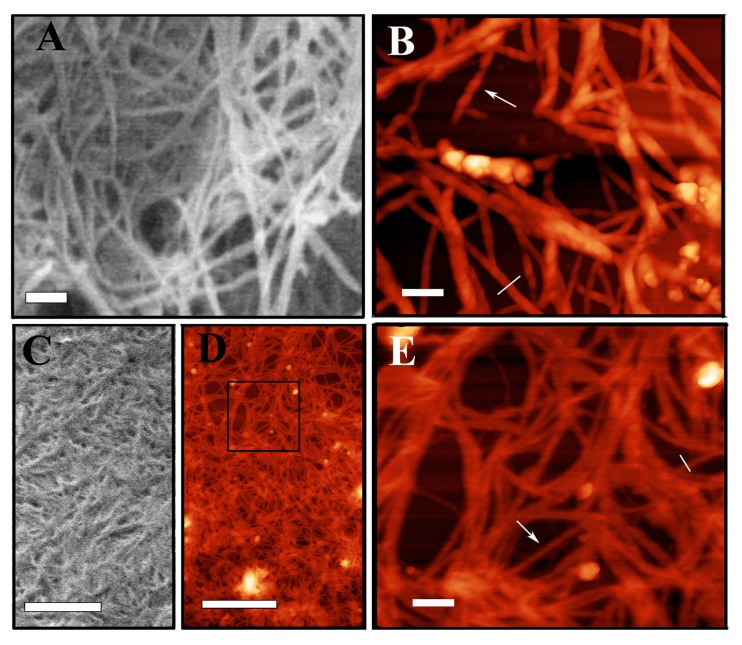

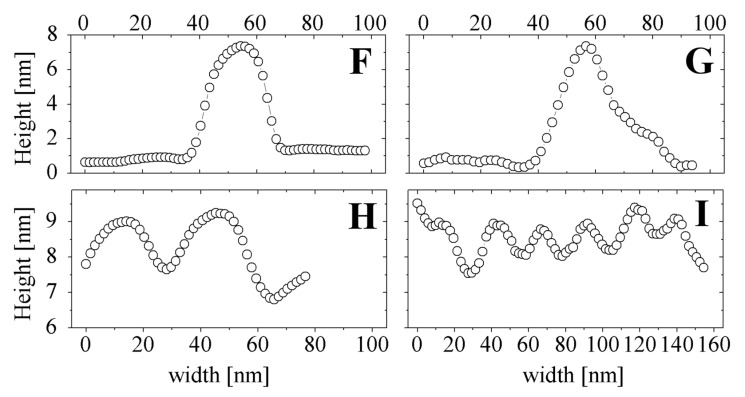
Morphology of FmocF*F*F* (**A**-**B**) and FmocF*F*F* crosslinked with 0.25 mM genipin (**C**-**D**-**E**) obtained by SEM (**A**-**C**) and AFM (**B**-**D**-**E**) microscopy. Panel **E** shows a magnification of the region delimited by a square in panel **D**. Bars in panel **A**-**C**-**D** correspond to 1 μm, in panels **B** and **E** to 100 nm. Panels **F** and **G** show the height profile of the fibers of FmocF*F*F* and FmocF*F*F* crosslinked with genipin, respectively, measured along the sections individuated by a continuous line in panels **B** and **E**. Arrows in panels **F** and **G** indicate fibers where twisting is visible, whose longitudinal section is reported in panel **H** and **I**.

SEM and AFM were employed for the investigation of the morphology of fibers and hydrogels, also in the presence of genipin. In these studies, we chose to focus on d-peptide based hydrogels, on the basis of their promising characteristics, also evidenced by our previous works [20]. Figure 8 shows the morphology of FmocF*F*F* alone and crosslinked with genipin obtained by SEM and AFM. Such investigations revealed that all the peptidic hydrogels self-assemble with similar features, also in the presence of chemical crosslinking, giving rise to nanofibers with similar structure. However, the presence of genipin seems to afford a different three-dimensional arrangement of the fibers, that results in an increase of fiber density. Also, in such conditions, the number of interconnections and knots between different fibers appears to increase, thus contributing to a more entangled organization of the hydrogel scaffold. In all the selected images, the fibers seem to be rather uniform in morphology and highly interconnected with knots. AFM analysis of the size of the fibers, both in the presence and in the absence of genipin, gives a narrow size distribution, with the same size, measured in the vertical direction of AFM images, of approximately 8 nm. Interestingly, in both hydrogels the R-handed twist repeats along the fiber length is visible (see, for example, the fibers marked by an arrow in panels B and E). The fiber pitch measured for FmocF*F*F* seems to be larger in respect to that of the same hydrogel crosslinked with genipin (around 50 nm and 30 nm, respectively, as determined from longitudinal profiles shown in corresponding panels H and I). This structural feature has to be further investigated in genipin-crosslinked FmocF*F*F* because it is observed with a minor clarity than in the non-crosslinked hydrogel, probably due to a variation of the imaging quality during the AFM scan.

## 3. Conclusions

Fmoc-Phe_3_ based hydrogels of different chirality prepared by using a biocatalytic approach have been chemically crosslinked with genipin. SEM and AFM investigations revealed that the peptidic hydrogels crosslinked with genipin are porous with highly entangled fibers. We also studied and compared some chemico-physical features of both crosslinked and non-crosslinked hydrogels obtaining biomaterials with different elastic modulus G'. Moreover, DXM encapsulation into the hydrogel seems to provide a more efficient and sustained release over time, making the system appealing for drug delivery approaches.

Overall, the results of these studies indicate that FmocF*F*F*–genipin hydrogels may be a useful scaffold for a variety of tissue engineering applications. We are currently attempting to discern the mechanisms of genipin crosslinking and determine the *in vitro* and *in vivo* cell attachment and degradation rate of genipin-crosslinked hydrogels.

## 4. Experimental Section

### 4.1. Materials

Fluorenylmethyloxycarbonyl-l-phenylalanine (FmocF) (>99%), Fluorenylmethyloxycarbonyl-d-phenylalanine (FmocF*) (>99%), l-diphenylalanine (FF) (>99%) and d-diphenylalanine (F*F*) (>99%) were purchased from Bachem GmbH (Weil am Rhein, Germany). Lipase from *Pseudomonas fluorescens* (PFL) (≥20.000 U/mg), genipin (≥98%) and all other chemicals were purchased from Sigma Aldrich (St. Louis, MO, USA) and used without further purification. All solvents used in HPLC analysis were of HPLC grade and used as received.

### 4.2. Biosynthesis of Peptide Hydrogels

F-moc tripeptide hydrogels of different chirality (FmocFFF and FmocF*F*F*) were prepared as previously reported [20]. Briefly, 40 μmol of each substrate, an Fmoc-aminoacid and a dipeptide, were suspended in a mixture of 1 mL of H_2_O and 420 μL of 0.5 M NaOH and magnetically stirred until obtaining a homogeneous dispersion. Then, 0.1 M HCl was added to reach a final pH value of 7. A fixed amount (100 μL) of enzymatic solution (50 mg/mL) was then added to the substrate suspension and the mixture was placed in a thermostated bath at 30 °C for 30 min.

Crosslinked hydrogels were prepared by following a similar procedure, adding to the substrate suspension at pH 7, before the enzymatic solution, a fixed amount (100 μL) of genipin solution with the selected concentration (0.1, 0.25, 0.5, 1, 5 and 10 mM).

Tripeptide final reaction yields were calculated indirectly by measuring Fmoc–Phe amino acid disappearance in the reaction medium. The reaction products obtained from the biosynthetic process were analyzed 24 h after their preparation. Samples were dissolved in a fixed volume of organic solution (60% ACN, 40% H_2_O, 0.1% TFA). 0.5 M NaOH was also added to a final pH value of 8. The solution was then centrifuged at 14.000 rpm for 20 min at constant temperature (25 °C). Naphthalene was added to the supernatant as the internal standard. HPLC measurements of the final Fmoc–Phe amino acid in the reaction mixture were performed by using the following experimental conditions: C-18 silica column, eluent: 60% ACN, 40% H2O, 0.1% TFA, flow rate: 0.9 mL/min, λ = 256 nm.

### 4.3. Rheological Measurements

The viscoelastic behaviour of hydrogels was studied by monitoring the storage and loss moduli, G' and G", using an AR2000 rheometer (Waters—TA Instruments, Milan, Italy) equipped with a parallel plates geometry (diameter 20 mm, gap 1 mm) with a fixed plate equilibrated at 25 °C. The mechanical spectra were obtained recording G' and G" in oscillatory mode, from 0.01 to 100 Hz, at constant strain of 1% (limit of the linear viscoelastic strain was about 10%). Kinetics of hydrogel formation at 30 °C were carried out by monitoring the time dependence of G' at 30 °C, at the constant frequency of 1 Hz.

### 4.4. Swelling and Stability Studies

The swelling ratios of Fmoc hydrogels in PBS (pH 7.4) were measured by adding to each hydrogel sample 3 mL of PBS and incubating it overnight at 30 °C. Fully swollen hydrogels were weighed (*W*_s_) immediately after the removal of excess water. Then, the hydrogels were freeze-dried and weighed again (*W*_d_). The swelling behavior was expressed, according to Equation (1), as the swelling ratio *q*, that is the ratio between the weight of the swollen sample (*W*_s_) and the weight of the freeze-dried hydrogel (*W*_d_)

*q* = (*W*_s_ − *W*_d_)/*W*_d_ (1)

Each experiment was performed in triplicate.

Hydrogel *in vitro* degradation was evaluated by adding to previously synthesized Fmoc hydrogels a fixed amount (8.5 mL) of Ringer solution (NaCl 8.6 mg/mL, KCl 0.3 mg/L. CaCl_2_ 0.33 mg/mL) and placing the system in a thermostated bath at 37 °C for 30 days. Hydrogels were weighed before the addition of the Ringer solution (*W*_0_) and after its removal (*W_t_*) and the weight loss ratio (Δ*W*) was calculated as:

Δ*W* = (*W*_0_ − *W_t_*)/*W*_0_ (2)

### 4.5. DXM in Vitro Release Experiments

DXM-loaded hydrogels, containing 6 mg of drug, were prepared by adding DXM-loaded PLGA or CS-coated PLGA NPs, or the corresponding amount of free DXM, during hydrogel formation. DXM-loaded PLGA or CS-coated PLGA NPs were prepared by a patented methodology [23], as described in previous works [24]. The mixture containing hydrogel precursors and free or entrapped DXM was then incubated at 30 °C for 1 h for gelation. Self-supporting hydrogels, entrapping DXM in their network, were formed in such conditions. After their preparation, DXM-loaded hydrogels were incubated in 2 mL of PBS (pH 7.4, 0.1 M) at 37 °C using a thermostated bath. At fixed time intervals, the supernatant was withdrawn and substituted with an equal amount of fresh PBS. DXM concentration in the supernatants collected at different time points were determined by HPLC by using the following experimental conditions: C-18 silica column, eluent: 60% ACN, 40% H_2_O, 0.1% TFA, flow rate: 0.9 mL/min, λ = 243 nm.

### 4.6. SEM and AFM Measurements

Hydrogel morphology was investigated by SEM and AFM microscopy. SEM images were collected by using a Zeiss Auriga 405 microscope at low extracting voltage (1.5–4 kV) and current (7.5 m aperture), in order to reduce the significant charging of the substrate and avoid radiation damages and artifacts [25], and at a very low working distance (≈1 mm) to improve the quality of the signal received by the in-lens detector. Hydrogel samples were cryo-fractured. Internal fragments were collected, freeze-dried, and mounted on an aluminum stab using double-sided carbon tape.

AFM images of the peptidic hydrogels were acquired in air at room temperature using a Dimension Icon (Bruker AXS, Billerica, MA, USA) instrument operating in Scan Asyst™ mode, with dedicated probes and using an ultra-sharp silicon tip (nominal radius of curvature 2 nm). This imaging mode allows the application of lower forces than standard tapping mode. Sample preparation for AFM measurements was performed according to the protocol described in a previous paper [20]. Aliquots of 10–20 μL were removed from the peptide hydrogel sample at the end of the gelation process and deposited onto a freshly cleaved mica surface. To optimize the amount of peptide adsorbed, each aliquot was left on mica for 5 min and then gently washed with 200 μL of Milli-Q water. The mica surface with the adsorbed peptide was then flushed with a stream of nitrogen for drying, and analyzed after 30 min. Images were analyzed using the Gwiddion free software and are presented as raw data, except for flattening. No further image processing was carried out.
